# Abnormal expression of lysosomal glycoproteins in patients with congenital disorders of glycosylation

**DOI:** 10.1186/s13104-023-06314-1

**Published:** 2023-04-17

**Authors:** Sahar Sabry, Noura R. Eissa, Maha S. Zaki

**Affiliations:** 1 Biochemical Genetics Department, Human Genetics and Genome Research Institute, National Research Centre (NRC), Cairo, Egypt; 2 Medical Molecular Genetics Department, Human Genetics and Genome Research Institute, National Research Centre (NRC), Cairo, Egypt; 3 Clinical Genetics Department, Human Genetics and Genome Research Institute, National Research Centre (NRC), Cairo, Egypt

**Keywords:** Congenital disorders of glycosylation, Total leukocytes, Lysosomes, Lysosome-associated membrane, Cystinosin, Cathepsin C

## Abstract

The study of the impact of some inherited defects in glycosylation on the biosynthesis of some lysosomal glycoproteins. Results description: Whole-exome sequencing revealed a homozygous variant; 428G > A; p. (R143K) in *SRD5A3* in one patient and a heterozygous one c.46G > A p. (Gly16Arg) in *SLC35A2* in the other patient. Both variants were predicted to be likely pathogenic. Lysosome-associated membrane glycoprotein 2 (LAMP2) immunodetection in both cases showed a truncated form of the protein. Cystinosin (CTN) protein appeared as normal and truncated forms in both patients in ratios of the mature to truncated forms of CTN were lower than the control. The levels of the truncated forms of both cellular proteins were higher in the SRD5A3-CDG case compared to the SLC35A2-CDG case. The tetrameric form of cathepsin C (CTSC) was expressed at low levels in both cases with congenital disorder of glycosylation (CDG). SLC35A2-CDG patient had one extra-unknown band while SRD5A3-CDG patient had a missing band of CTSC forms. The expression patterns of lysosomal glycoproteins could be different between different types of CDG.

**Supplementary Information:**

The online version contains supplementary material available at 10.1186/s13104-023-06314-1.

## Introduction

Glycosylation is the major post-translational modification of proteins. Glycoproteins play pivotal roles in many biological processes that take place at the cellular, tissue, and whole-organism levels [1]. Among these biological processes are some lysosomal functions. The activity and stability of many lysosomal glycoproteins are regulated by their glycosylation [2]. The lysosomal glycoproteins perform a protective function where the inner side of the lysosomal membrane is covered with heavily glycosylated proteins, known as lysosomal-associated membrane proteins (LAMPs). They protect the lysosomal membrane from self-degradation by the lysosomal hydrolases [3]. Congenital disorders of glycosylation (CDG) are a large class of inborn errors of metabolism. More than 150 types of CDG have been identified. They result from inherited defects in the glycosylation-mediators such as enzymes, chaperones, transporters, etc. [4]. CDGs are classified into CDG type I and type II. CDG type I results from a defective biosynthesis of the glycan chain and/or its transfer on to the protein in the rough endoplasmic reticulum (ER). CDG type II results from an impaired processing and modification of the glycan chain linked to protein [5, 6]. The clinical spectrum of CDGs is large; it could involve one-system manifestations or multiple systems involvement [7].

**Fig. 1:**
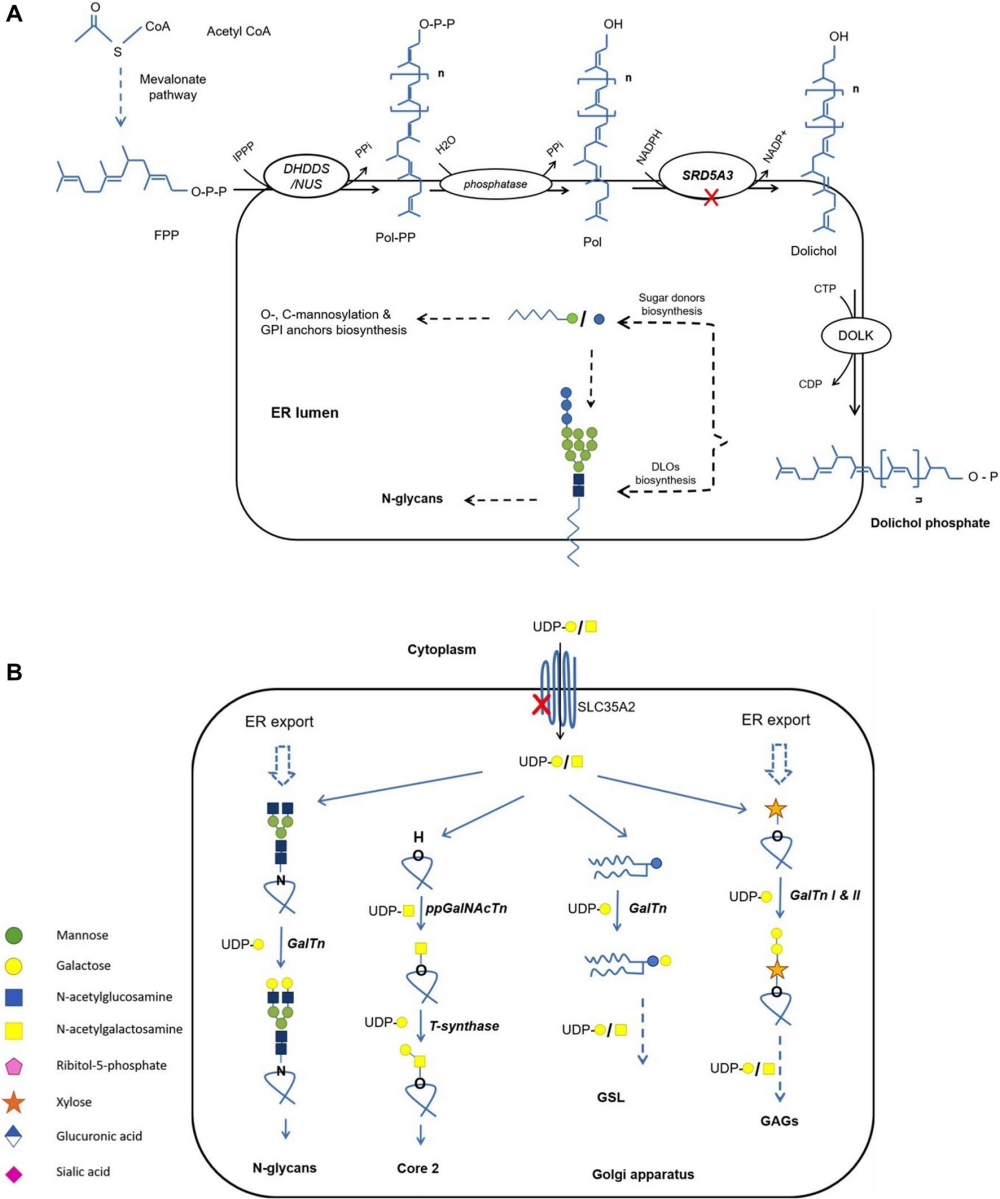
A diagram showing the location of the defects (the red marks) in the glycosylation pathways in both CDG cases. **A** The mevalonate pathway and biosynthesis of dolichol on the cytosolic side of the ER and the subsequent glycosylation steps. **B** UDP-Galactose translocation from the cytoplasm into the GA and its involvement in the galactosyltransferases-mediated galactosylation of glycoconjugates

Congenital disorders of glycosylation type I include defects in the biosynthesis of a lipid molecule known as dolichol. In 1970, Behrnes and Leloir have described the role of dolichol phosphate in the glycosylation process [8]. It acts as a carrier of the growing glycan chain as well as some sugar donors in the N-glycosylation process that takes place in the ER [9]. In the dolichol biosynthesis pathway, an ER-bound steroid 5-alpha-reductase 3 (SRD5A3) reduces the terminal double bond (α isoprene unit) in polyprenol and converts it to dolichol in a NADPH-dependent reaction [10–12]. SRD5A3 is a rate-limiting enzyme in the biosynthesis pathway of dolichol, which then is activated into dolichol phosphate (Dol-P) [10] (Fig. 1A). Mutations in *SRD5A3* results in SRD5A3-CDG (formerly classified as CDG type Iq) (OMIM 612379). The clinical picture involves mental retardation, brain malformation, and skin manifestations such as ichthyosis and skin hyperpigmentation.

Congenital disorders of glycosylation type II result from the incomplete modifications of the glycans on proteins after their export from the ER to the Golgi apparatus. These modifications involve sequential enzymatic removal and addition of monosaccharides in a process that takes place exclusively in the Golgi apparatus [13, 14]. The glycosylation machinery in the Golgi apparatus involves some specific nucleotide sugars transporters. Solute carrier 35 member A2 (SLC35A2) is UDP-Galactose transporter that is bound to the Golgi apparatus membrane. It translocates the UDP-Galactose from the cytoplasm to the Golgi apparatus lumen, wherein galactose is transferred by galactosyltransferases during the modification of the N- and O-glycans as well as glycosphingolipids biosynthesis (Fig. 1B). Mutations in *SLC35A2* result in SLC35A2-CDG (formerly classified as CDG IIm) (OMIM 300896). Patients with SLC35A2-CDG present with dysmorphic features, brain abnormalities, and seizures.

This study aims to study the impact of two novel mutations in two genes that are involved in the N-glycan biosynthesis as well as its modification on the expression profile of three lysosomal glycoproteins; each one of them has a different function inside the lysosome. One glycoprotein is in the glycocalyx of the lysosome (Lysosomal membrane associated protein, LAMP2) with a protective function. The second glycoprotein  is a lysosomal transmembrane transporter (Cystinosin, CTN), which transports cysteine out of the lysosomes. The third glycoprotein is a lysosomal enzyme (Cathepsin C, CTSC) that undergoes several activation steps in the Golgi apparatus and lysosomes before being secreted in the exosomes.

To our knowledge the studied lysosomal glycoproteins have not been studied before neither in patients with SRD5A3-CDG nor SLC35A2-CDG.

## Subjects and methods

### Subjects

The Research Ethics Committee of the National Research Centre according to the “World Medical Association Declaration of Helsinki” approved this study. The study protocols were followed in accordance with Declaration of Helsinki. Written informed consent was obtained from the patient’s legal guardians.

### Clinical history of the studied cases

**Fig. 2:**
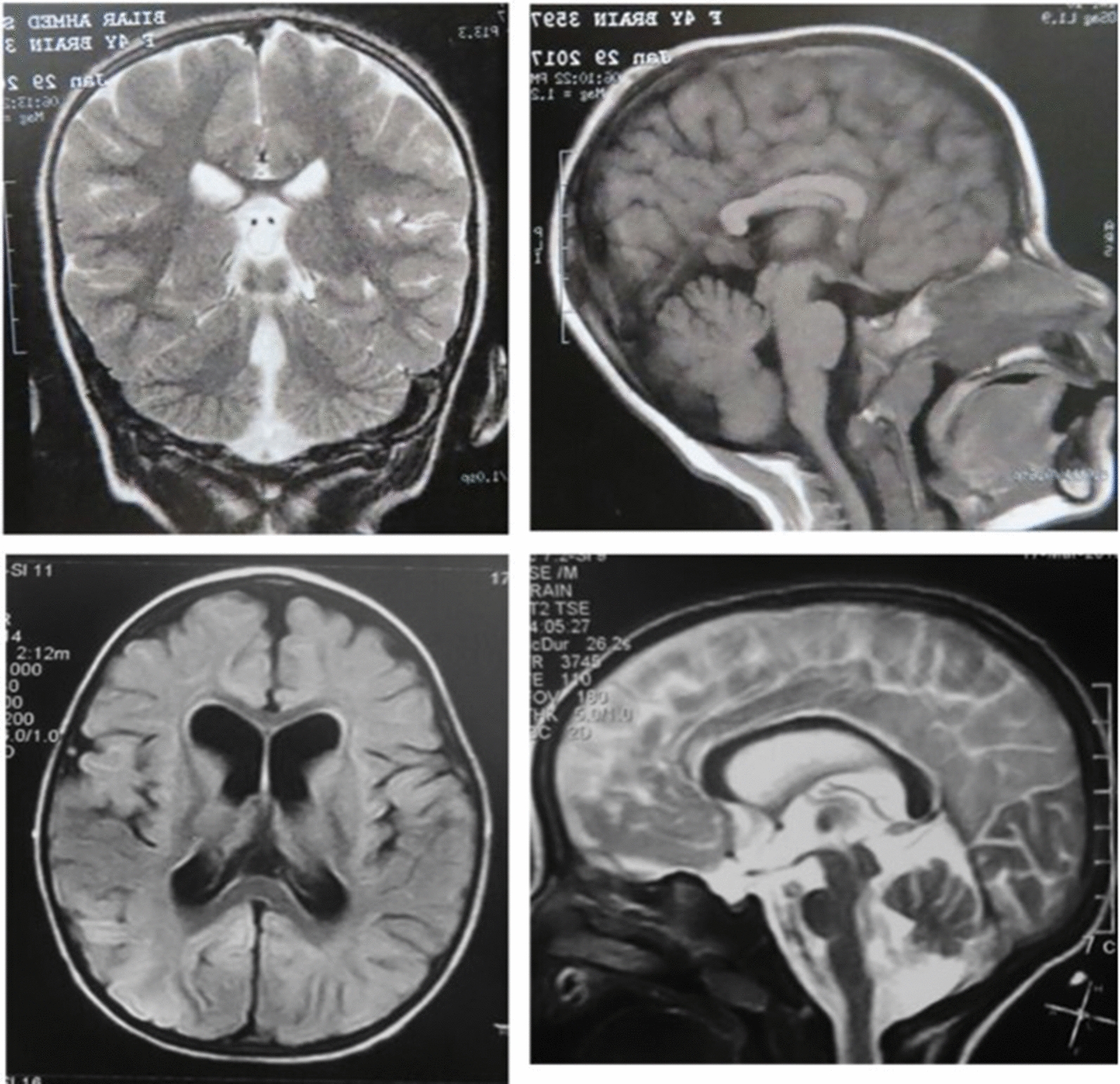
MRI images of the studied CDG cases. Top images: The SLC35A2-CDG patient showing a short malformed corpus callosum and an inferior vermis hypoplasia. Bottom images: The SRD5A3-CDG patient showing a mild prominent cortical sulci frontally dilated lateral ventricles and a significant reduction of vermian size

Two female patients were included in this study. The pregnancy and delivery histories were uneventful in both cases. MRI (Fig. 2) fundus examination, and EEG (electroencephalogram) were done. Other investigations (including metabolic screening, echocardiography, and abdomen sonar) were normal in both patients. The clinical presentation and preliminary investigations of both patients are presented in Table 1.

**Table 1:** Clinical and laboratory investigations of the studied CDG cases

	**SLC35A2-CDG**	**SRD5A3-CDG**
Age at diagnosis	3 year	1 10/12 year
Milestones	Delayed	Delayed
Neurological assessment	Hypotonia with preserved reflexes Good sensation and coordination	Axial hypotonia Limb hypertonia Grasped hands Brisk reflexes
Fundus examination	Normal	Bilateral optic nerve hypoplasia, mottle fundus Marked reduced scotopic in both eyes Bilateral low amplitude in VEP Rotatory nystagmus
Anthropometric measurements	Weight 12.5 kg (− 1.5SD), Length 85 (− 2.1SD) and Head circumference 45 cm(-3.4SD)	Weight 9.1 kg (− 2.3SD) Length 82 cm (− 0.5SD) Head circumference 44.5 cm (− 2.5SD)
MRI	Short malformed corpus callosum Inferior vermis hypoplasia (Fig. 2, top images)	Mild prominent cortical sulci frontally dilated lateral ventricles Abnormal white matter signal at T-FLAIR Significant reduction of vermian size (Fig. 2, bottom images)
EEG	Bitemporal epileptogenic activity	Bilateral temporoparietal epileptogenic discharge
Facies	Long face Narrow forehead Straight and down slanting eyebrows Synophrys Depressed nasal bridge Bulbous nasal tip Long philtrum Straight lips Flat chin Low set ears with folded helix	Long face Trichomegaly Prominent nose Low set large ears
Bone	Delayed bone age. The hands showed single transverse palmar creases and hypoplastic distal phalanges of the 5th finger bilaterally	The left congenital hip dislocation was identified at the age of 8 months
Serum transferrin isoelectric focusing	Increased mono-, di-, and tri-sialotransferrin (Additional file 1: Figure S1)	Increased asialyl-, mono-, di-, and tri-sialotransferrin (Additional file 1: Figure S1)

## Methods

### Whole exome sequencing (WES)

Whole exome sequencing was performed as a trio for each family's affected member and parents. All variants were prioritized by allele frequency, conservation, and predicted effect on protein function. Double-stranded DNA capture baits against approximately 36.5 Mb of the human coding exome (targeting > 98% of the coding RefSeq from the human genome build GRCh38/hg19) are used to enrich target regions from fragmented genomic DNA with the Twist Human Core Exome Plus kit. The generated library is sequenced on an Illumina platform to obtain at least 20 × coverage depth for > 98% of the targeted bases.

All disease-causing variants were reported in HGMD^®^, in ClinVar, and dbSNP^®^ as well as all variants with minor allele frequency (MAF) below 1% in the gnomAD database were considered. The investigation for relevant variants was focused on coding exons and flanking ± 20 intronic bases. All potential modes of inheritance patterns are considered. Variant filtration and selection considered the provided family history and clinical information and were used to evaluate identified variants concerning their pathogenicity and causality. Variant’s pathogenicity/deleteriousness were evaluated by their using online tools PolyPhen_2 (http://genetics.bwh.harvard.edu/pph2), SIFT (https://sift.bii.a-star.edu.sg/www/Extended_SIFT_chr_coords_submit.html), and Mutation Taster (http://www.mutationtaster.org) [15].

### Total leukocytes isolation and protein extraction

Five mL of peripheral blood were collected on EDTA from the control subjects and the patients. Leukocytes were isolated by lysing RBCs using ammonium chloride [16]. Proteins were extracted from the cells using a lysis buffer containing 0.1% phenylmethylsulfonyl fluoride (PMSF) (Sigma, USA). The extracted proteins were quantified using the BCA protein assay kit (Thermo Scientific, US).

### Immunoblotting of lysosomal glycoproteins

Fifty micrograms of protein extracted from leukocytes or 0.5 µL plasma, were loaded onto 4 –12% SDS-PAGE gels (Invitrogen, Thermo Scientific, US). After electrophoresis, proteins were transferred onto nitrocellulose membranes (Amersham, USA). The membranes were saturated for 30 min with 5% fat-free milk in TBST (Tris buffer saline with 0.1% Tween 20), and then they were incubated with an optimized dilution of the primary antibody overnight. The next day, the blots were incubated for an hour with the secondary antibodies. Protein bands were detected by a chemiluminescence revelation kit (Novex, Invitrogen, Thermo Scientific, US) and a CDD camera was used to detect the bands. The primary antibodies used: were mouse LAMP2 antibody (Novus Biologicals, USA), mouse cystinosis antibody (Santa Cruz, USA), β-actin antibody, mouse GAPDH antibody, and mouse anti-cathepsin C antibody (Santa Cruz, USA). Anti-mouse secondary antibody (Santa Cruz, USA) was used to probe the primary antibodies.

## Results

### The identified variants by WES

**Fig. 3:**
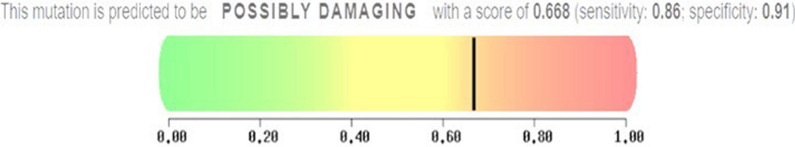
PolyPhen-2 v2.2 prediction score for *SLC35A2* variant, c.46G > A p. (G16R)

*Patient 1* a heterozygous variant ChrX (GRCh38.p12): g.48768868C > T,NM_001282651: c.46G > A; p. (G16R) in *SLC35A2* (ENST00000247138.10; MIM: 314375) was detected. Both parents showed wild type for the corresponding variant. Thus, suggesting a de Novo variant causing the autosomal dominant X-linked congenital disorder of glycosylation SLC35A2-CDG. This variation mutates the first exon of *SLC35A2*. The defect is located in the first transmembrane helical domain. PolyPhen-2 (v2.2) prediction score was 0.668 as possibly damaging variant. Variant Taster predicted that protein features might be affected; yet the variant is benign (Fig. 3). On the other hand, SIFT tool report showed that Substitution at pos 16 from G to R is predicted to “affect protein function” with a score of “0.00”, Median sequence conservation: 4.32, and Sequences represented at this position1 (Additional file 1: Fig. S2). The Allele ‘T’ was not found in ExAC, 1000G, or gnomAD. This variant is classified to be likely pathogenic according to the ACMG guidelines [17].

**Fig. 4:**
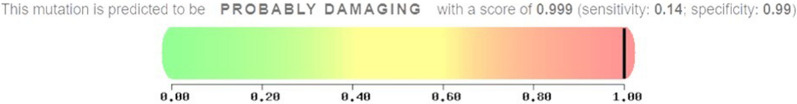
PolyPhen-2 v2.2 prediction score for *SRD5A3* variant, c.428G > A p. (R143K)

*Patient 2* a homozygous variant in *SRD5A3* (ENST00000264228.8; MIM 611715) was identified in exon 3; Chr4 (GRCh38.p12): g.56230304G > A, NM_024592.4: c.428G > A; p (R143K). Both parents were carriers of the same variant. This site is a conserved site of the protein and represents a starting point for a naturally occurring isoform of the protein. It runs through amino acids 143–396. Accordingly, any change in this amino acid might disrupt the function of the entire protein (https://pubmed.ncbi.nlm.nih.gov/14702039/). PolyPhen-2 (v2.2) prediction score was 0.999 and the variant is predicted to be a probably damaging one. Variant Taster predicted this variant as a deleterious variant (Fig. 4). The SIFT tool report showed that Substitution at pos 143 from R to K is predicted to affect the protein function with a score of 0.00, Median sequence conservation: 3.38, and Sequences represented at this position 6 (Additional file 1: Fig. S3). This variant was not found in Clinvar, ExAC, 1000G, or gnomAD. According to the ACMG guidelines; it is classified to be likely pathogenic [17].

### The atypical pattern of lysosomal glycoproteins

**Fig. 5:**
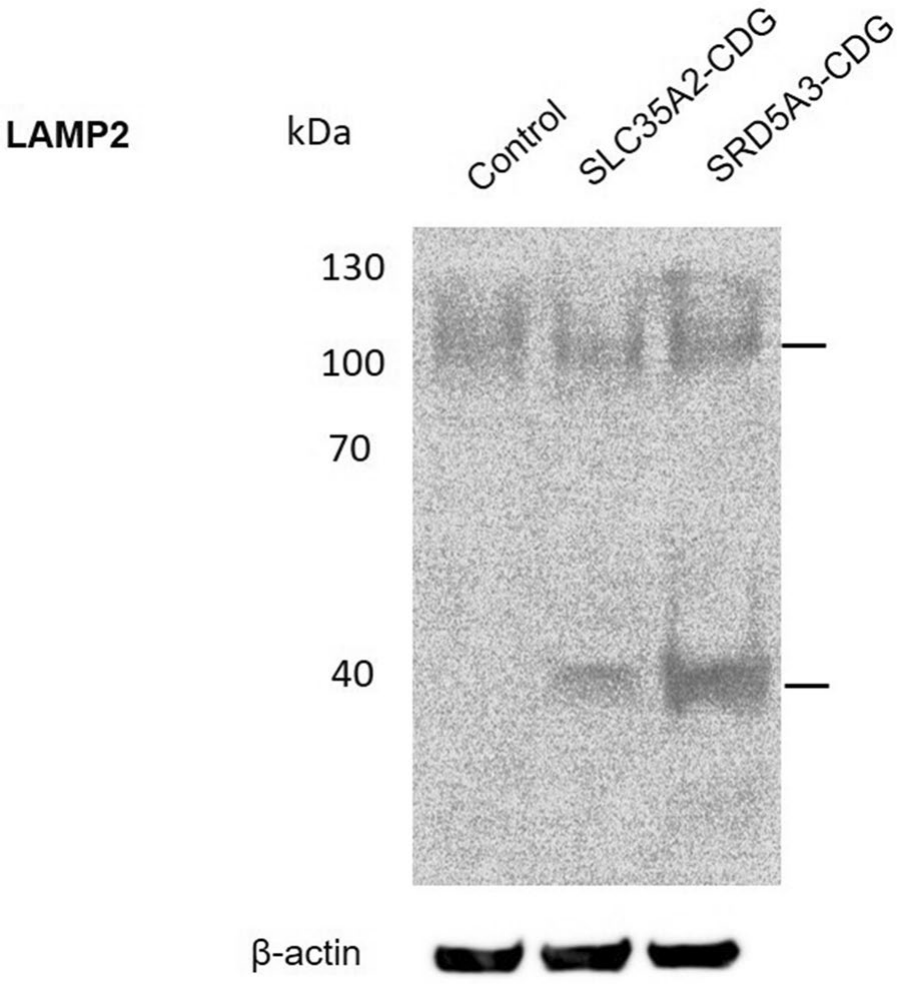
Immunoblotting of LAMP2 protein in total leukocytes separated on an SDS-PAGE gel. Beta-actin was used as a housekeeping protein

*Lysosome-associated membrane glycoprotein 2 (LAMP2)* The control showed one diffuse band at an approximate molecular weight (MW) of 100–120 kDa. Both patients showed two bands. One band at MW 100–120 kDa appeared less diffuse compared to the control sample. Another band with MW ~ 40 kDa was found in both patients and it was absent in the control sample. The intensity of the truncated form of LAMP2 in the SRD5A3-CDG patient’s sample was higher than the SLC35A2-CDG’s one (Fig. 5). Quantification of the intensities of the bands was done using image J software (Additional file 1: Fig. S4).

**Fig. 6:**
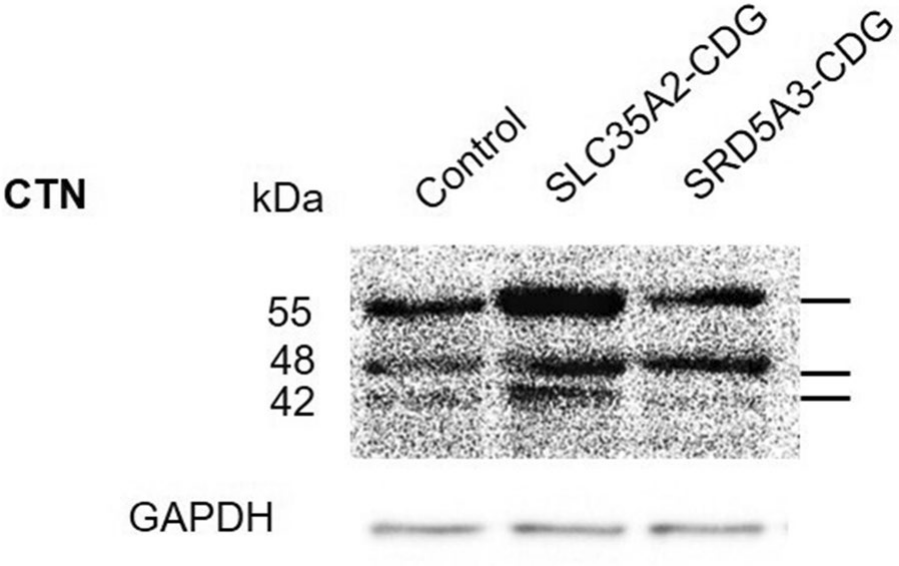
Immunoblotting of CTN protein in total leukocytes separated on an SDS-PAGE gel. GAPDH was used as a housekeeping protein

*Cystinosin (CTN)* The control subject and both patients showed three bands at MW 55, 48, and 42 kDa. The three bands had different relative intensities in each sample. The control sample showed the 55 kDa band with an intensity higher than the other two bands, which had an almost equal intensity. The SLC35A2-CDG patient had an increased intensity of all the bands with the highest one being with MW 55 kDa. SRD5A3-CDG patient had almost equal intensities of 48 and 55 kDa bands and a relatively reduced intensity of the 42 kDa band (Fig. 6). Quantification of the intensities of the bands was done using image J software (Additional file 1: Fig. S5).

**Fig. 7:**
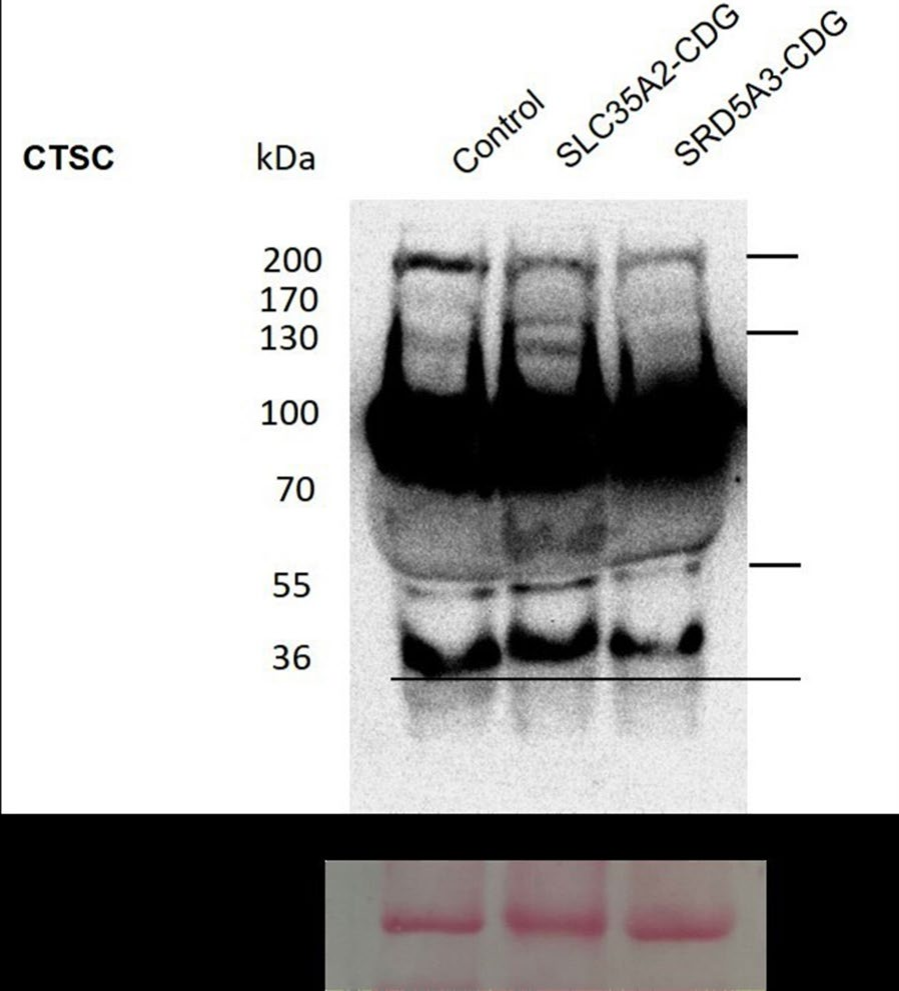
Immunoblotting of CTSC protein in plasma separated on an SDS-PAGE gel. Blot stained with ponceau S was used as a loading control

*Cathepsin C (CTSC)* the samples of the control and the patients had a similar intensity of the most expressed band with MW 100 kDa. The differences between the control and both patients were observed in four bands with MW 36, 50, 130, and 200 kDa. Both patients showed a reduced intensity of MW 200 kDa band. A band at MW 130 kDa was more intense in the SLC35A2-CDG patient and it was absent in the SRD5A3-CDG patient’s sample. SLC35A2-CDG patient’s sample showed an extra band at an approximate MW 150 kDa, which was absent in the control and the other patients' samples. A band at MW 50 kDa was with equal intensities in the control and SRD5A3-CDG patient and it was with higher intensity in the SLC35A2-CDG patient’s sample. A band with MW 36 kDa was found in the control sample while it was observed with a slightly increased MW (~ 41 kDa) in both patients’ samples (Fig. 7). Quantification of the intensities of the bands was done using image J software (Additional file 1: Fig. S6).

## Discussion

The current study is of a considerable value as to our knowledge this work involves the first study of both secreted (in plasma) and membrane-bound (derived from the patients’ white blood cells) lysosomal glycoproteins in two CDG patients with two novel mutations. Our study shows that the studied CDG cases had an abnormal expression, and or activation in some lysosomal glycoproteins.

Despite the presence of the novel variants, the two CDG cases described here showed a clinical presentation that was comparable to many of the previously reported cases with the same CDG-causing genetic defect [18, 19]. However, unlike most of the reported SRD5A3-CDG cases [20–22], the case described here did not have any clear skin manifestations. SRD5A3 enzyme is involved in almost all types of protein glycosylation; N-glycosylation, O-mannosylation, C-mannosylation, and GPI-anchors biosynthesis. This could explain the presence of multiorgan involvement as well as the higher severity of the clinical signs and symptoms compared to the SLC35A2-CDG patient in our study. Similarly, multiorgan deterioration was reported in CDG types resulting from defects along the dolichol biosynthesis pathway such as DOLK-CDG [23], and DHDDS-CDG [24]. Both mutations detected were novel and confirmed in both patients. The variant in *SLC35A2* was not found in any of the conserved domains of the protein. Yet, according to the ACMG 2015 guidelines, this mutation was classified as a likely pathogenic mutation based on the absence of population data, computational and predictive data, functional data, and being a De novo variant [17]. The variant in *SRD5A3* is mapped to a conserved domain of the protein. No other alterations have been reported to this position before, only a nonsense mutation one codon upstream (p.R142X) was reported in 2010 in a compound heterozygous pattern [11]. Since the arginine side chain contains more number of NH2 groups than Lysine;  the substitution of the amino acid arginine with lysine leads to a partial loss of the interactions and role of the side chain at this position. Along with the conserved nature of this domain, this alteration most probably would have deleterious effects on the function of the protein produced.

Lysosome-associated membrane glycoprotein 2 (LAMP2) and CTN are lysosomal membrane-bound glycoproteins and were assessed in total leukocytes, whereas CTSC secreted into the extracellular space in exosomes was analyzed in plasma. We found differences in the expression patterns of the normal and truncated forms between our CDG patients and the control.

The LAMP2 expression profile was the same in both CDG cases. They had one band of the normal form (MW 120 kDa) and another band of the truncated form (MW 40–45 kDa). The relative abundance of the normal and truncated forms in the SRD5A3-CDG patient was almost the same. Alternatively, the SLC35A2-CDG patient had a less abundant truncated form compared to the normal one. Unlike our patients, *TMEM165*-deficient cell lines and animals showed an accumulation of the truncated form only [25]. A profile similar to our patients’; with the presence of both forms of LAMP2, was obtained when *TMEM165*-deficient cells were supplemented with minerals and monosaccharides [25, 26]. Alternatively, the expression profile of LAMP2 was found normal in skin fibroblasts derived from a group of TMEM165-CDG patients [27] and with a relatively different migration pattern in a patient with ATP6P1-CDG [28]. This could be attributed to the difference in cell types in which LAMP2 expression profiles were analyzed. Total leukocytes could have some advantages over fibroblasts in detecting the mature and truncated forms of glycoproteins. Moreover, different glycosylation defects could result in different expression patterns of proteins.

Nevo et al. studied the impact of disrupted glycosylation on CTN turnover rate. They reported an increased degradation of CTN due to deletion of one of the N-glycosylation sites as well as PNGase F-induced removal of N-glycans [29]. The immunodetection of CTN in leukocytes derived from our CDG patients revealed an accumulation of the normal and truncated forms compared to the control**.** The observed truncated forms of CTN could be the products of the normal form turnover. Like the LAMP2 protein, the truncated form of CTN had almost the same abundance as the normal form in leukocytes derived from the SRD5A3-CDG patient. On the other hand, SLC35A2-CDG patient’s leukocytes showed a normal/truncated ratio that was comparable to the control.

The process of maturation/activation of CTSC is a multi-step process that takes place in the Golgi apparatus and the lysosomes [30]. CTSC protein had a relatively different expression profile in both CDG cases compared to the control. They showed reduced levels of the tetrameric form of CTSC (MW 200 kDa). SLC35A2-CDG patient plasma showed one extra band (MW 150 kDa) and an increased level of monomeric form (MW 52 kDa). An uncharacterized band (MW 130 kDa) was found in the control and SLC35A2-CDG patient samples but it was not found the in SRD5A3-CDG patient’s sample. The cathepsins L and S activation product of CTSC (MW 36 kDa) [30–32], was observed in the control sample and another band with higher molecular weight was found in both CDG patients. The dimeric form of the CTSC (MW 100 kDa) expression pattern was the same in both patients and the control. Treatment of synthetic and rat macrophage-purified CTSC with Endo H enzyme did not produce the same profile observed in our CDG patients’ plasma samples [33]. Hence, the observed missing and extra intermediates of CTSC could not be assumed to be some hypoglycosylated forms of the protein.

From these findings, one question could be raised: What is the underlying molecular mechanism that affects the expression of lysosomal proteins in patients with CDG type I and type II?

It has been observed that SRD5A3-CDG patient had different expression patterns compared to SLC35A2-CDG patient in the studied lysosomal glycoproteins. Here, the glycosylation process could be linked to the stability and bioavailability of the proteins. It could be assumed that a defect at the initial steps of the glycans biosynthesis; as in the case of mutant *SRD5A3*; would result in a complete loss of glycan chain which in turn could result in a faster rate of turnover of proteins when compared with a mutant *SLC35A2* in which there is a defect in the modification of the already synthesized glycan. This could explain the presence of more levels of truncated LAMP2 and CTN in leukocytes of SRD5A3-CDG compared with SLC35A2-CDG patient. Similarly, the glycosylation of proteins plays a pivotal role in their inter-organelles trafficking and this could explain the different patterns of the activation products of CSTC enzyme that takes place in the Golgi apparatus and the lysosome.

A remarkable feature of this study is that future attempts could be done to implement some lysosomal glycoproteins in leukocytes and plasma as new diagnostic biomarkers for congenital disorders of glycosylation as less invasive samples and informative assessment of glycoproteins compared to skin fibroblasts.

## Limitations of the work

The limitations of our work involve the number of studied cases as well as the number of investigated lysosomal glycoproteins. Additionally, further investigations are essential for the assessment of the consequences of the observed atypical expression of lysosomal glycoproteins on their biological functions.

## Supplementary Information

**Additional file 1: Figure S2.** SIFT tool prediction score for *SLC35A2* variant, c.46G>A p. (G16R). **Figure S3.** PolyPhen-2 v2.2 prediction score for *SRD5A3,* variant, c.428G>A p.(R143K). **Figure S1.** Isoelectric focusing (IEF) of serum transferrin. Standard Tf (lane 1), control (lane 2), SRD5A3-CDG patient (lane 3), and SLC35A2-CDG patient (lane 4). Strips were stained with Coomassie Blue. **Figure S4.** Histogram chart represents the quantified intensities of LAMP2 in total leukocytes bands obtained by western blot. The values were measured by image J software. **Figure S5.** Histogram chart represents the quantified intensities of CTN in total leukocytes bands obtained by western blot. The values were measured by image J software. **Figure S6.** Histogram chart represents the quantified intensities of bands of the activation products of CSTC enzyme in plasma obtained by western blot. The values were measured by image J software.

## Data Availability

All data generated or analyzed during this study are included in this published article and its Additional information files*.*

## Abbreviations list

CTSC: Cathepsin C
CDG: Congenital disorder of glycosylation
CTN: Cystinosin
EEG: Electroencephalogram
GA: Golgi apparatus
LAMP2: Lysosome-associated membrane glycoprotein 2
MAF: Minor allele frequency
MW: Molecular weight
SDS-PAGE: Sodium dodecyl sulfate–polyacrylamide
SLC35A2: Solute carrier 35 member A2
SRD5A3: Reticulum-bound steroid 5-alpha-reductase 3
WES: Whole-exome sequencing
