# Supplementary material for: Abnormal expression of lysosomal glycoproteins in patients with congenital disorders of glycosylation
Source: BMC Res Notes. 2023 Apr 17;16:53. doi: 10.1186/s13104-023-06314-1 (PMC10108535; doi:10.1186/s13104-023-06314-1)
Supplement: Supplementary file 1 — Additional file 1: Figure S2. SIFT tool prediction score for SLC35A2 variant, c.46G>A p. (G16R). Figure S3. PolyPhen-2 v2.2 prediction score for SRD5A3, variant, c.428G>A p.(R143K). Figure S1. Isoelectric focusing (IEF) of serum transferrin. Standard Tf (lane 1), control (lane 2), SRD5A3-CDG patient (lane 3), and SLC35A2-CDG patient (lane 4). Strips were stained with Coomassie Blue. Figure S4. Histogram chart represents the quantified intensities of LAMP2 in total leukocytes bands obtained by western blot. The values were measured by image J software. Figure S5. Histogram chart represents the quantified intensities of CTN in total leukocytes bands obtained by western blot. The values were measured by image J software. Figure S6. Histogram chart represents the quantified intensities of bands of the activation products of CSTC enzyme in plasma obtained by western blot. The values were measured by image J software. [file 13104_2023_6314_MOESM1_ESM.docx]

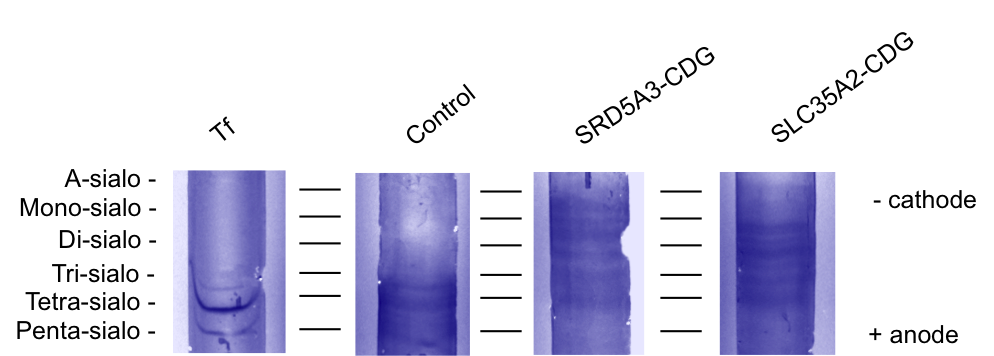


Fig. S1 Isoelectric focusing (IEF) of serum transferrin. Standard Tf (lane 1), control (lane 2), SRD5A3-CDG patient (lane 3), and SLC35A2-CDG patient (lane 4). Strips were stained with Coomassie Blue.


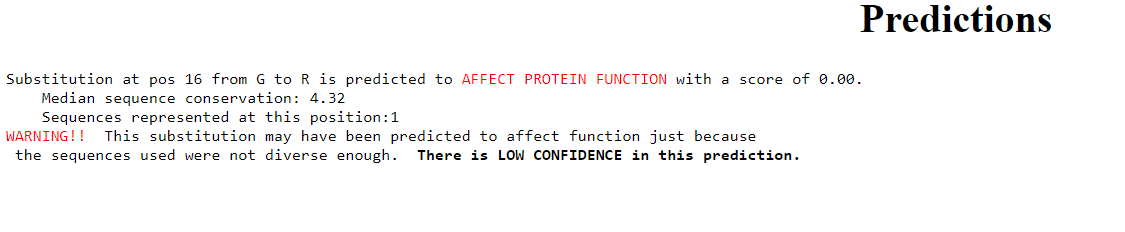


**Fig. S2** SIFT tool prediction score for *SLC35A2* variant, c.46G>A p. (G16R).


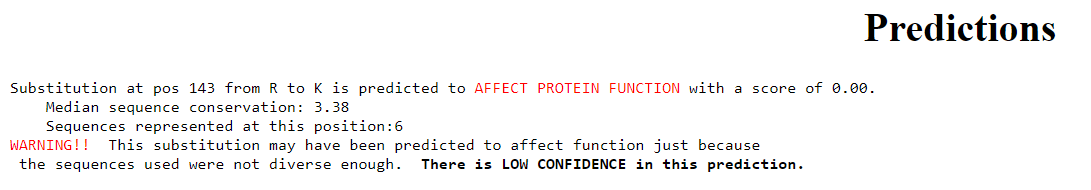


**Fig. S3** PolyPhen-2 v2.2 prediction score for *SRD5A3,* variant, c.428G>A p.(R143K).

**Fig. S4** Histogram chart represents the quantified intensities of LAMP2 in total leukocytes bands obtained by western blot. The values were measured by image J software.

**Fig. S5** Histogram chart represents the quantified intensities of CTN in total leukocytes bands obtained by western blot. The values were measured by image J software.

**Fig. S6** Histogram chart represents the quantified intensities of bands of the activation products of CSTC enzyme in plasma obtained by western blot. The values were measured by image J software.


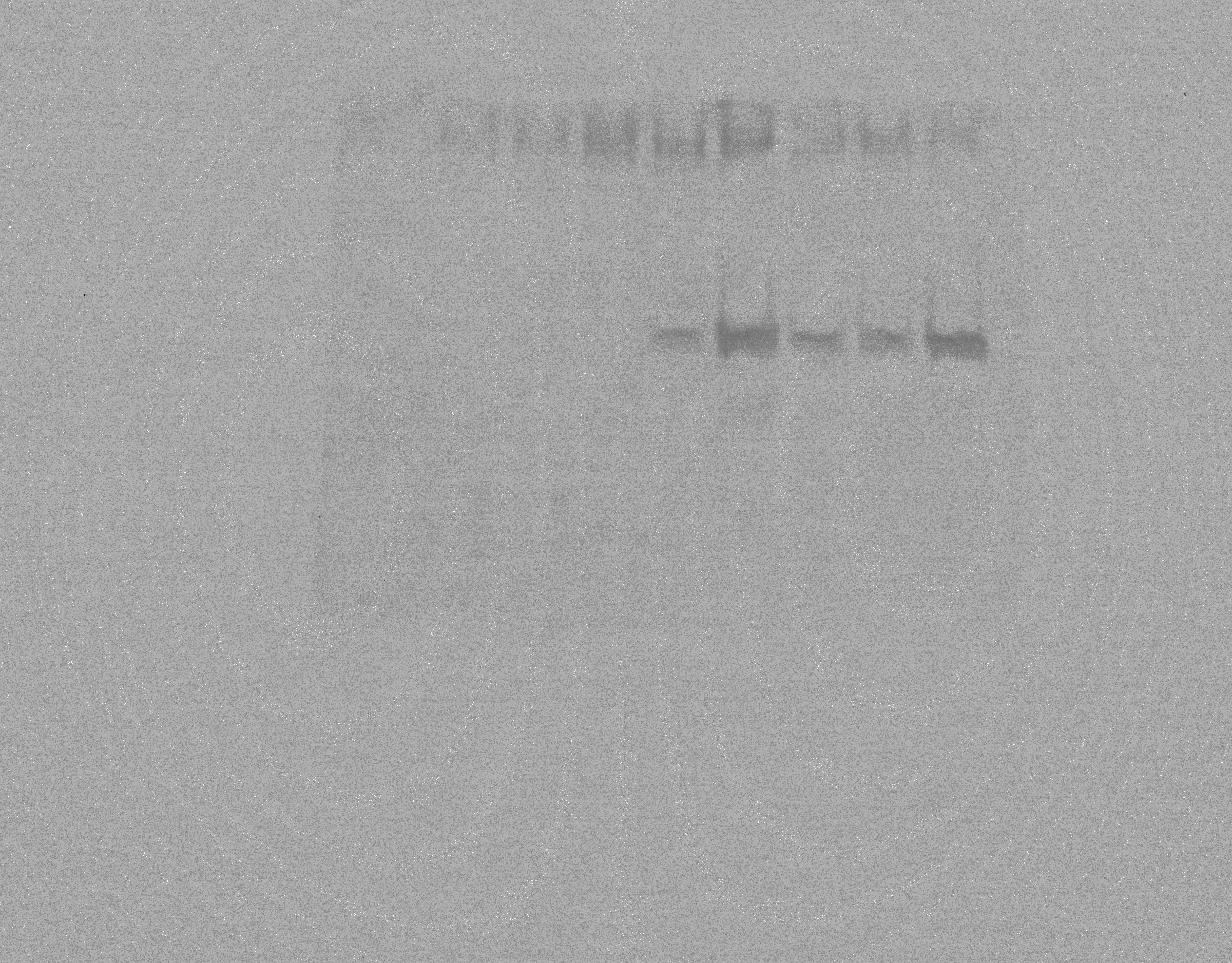


Control

SLC35A2-CDG

SRD5A3-CDG

The original blot of figure 5 showing LAMP2 protein expression profiles in a cohort of CDG patients. The red box is denoting the studied cases in our work. **The blot was cut and the beta actin was detected for only the studied cases.**

**CTN**

Control

SRD5A3-CDG

SLC35A2-CDG


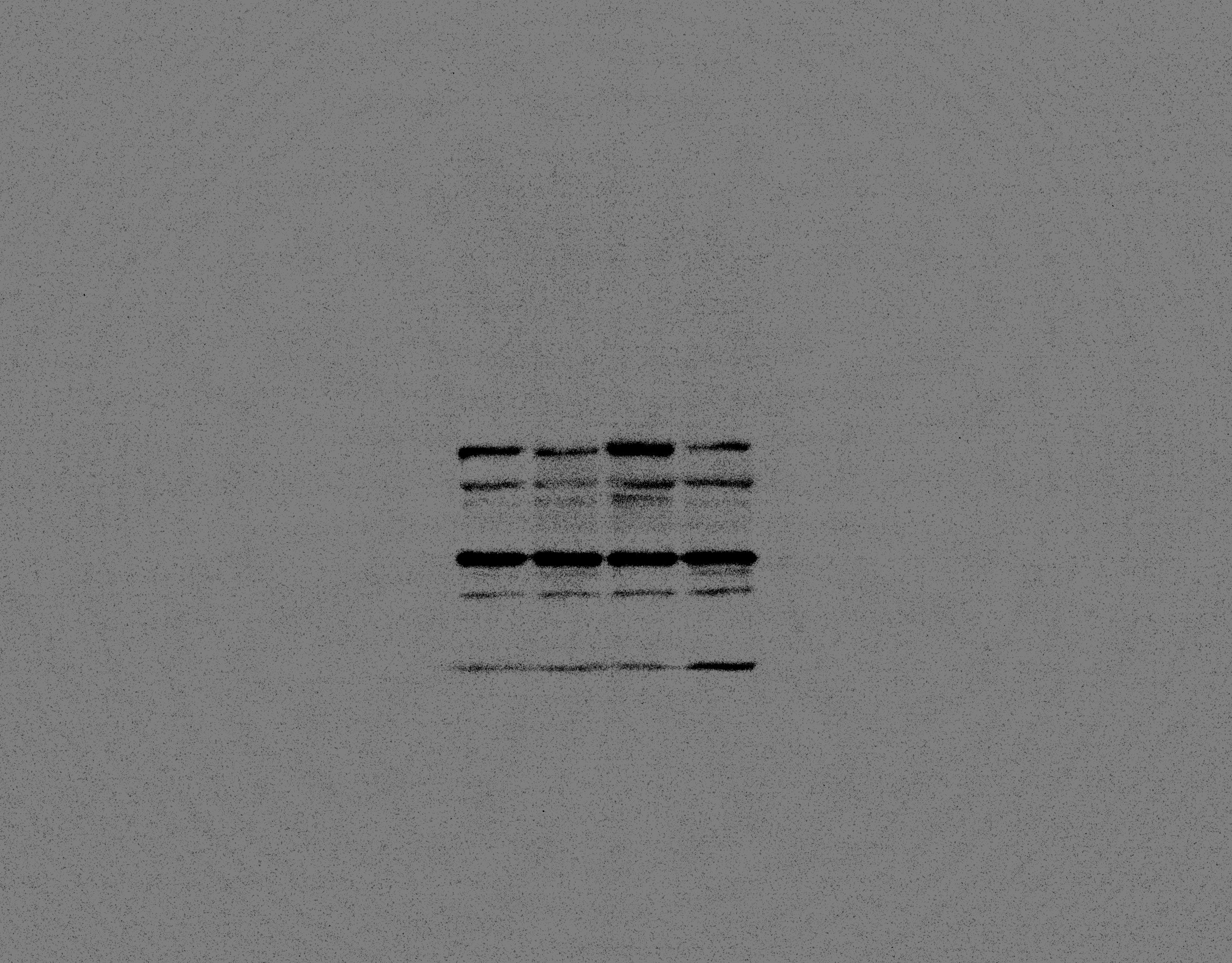


GAPDH

The original blot of figure 6 showing CTN protein expression profiles in CDG patients. The red box is denoting the studied cases in our work.


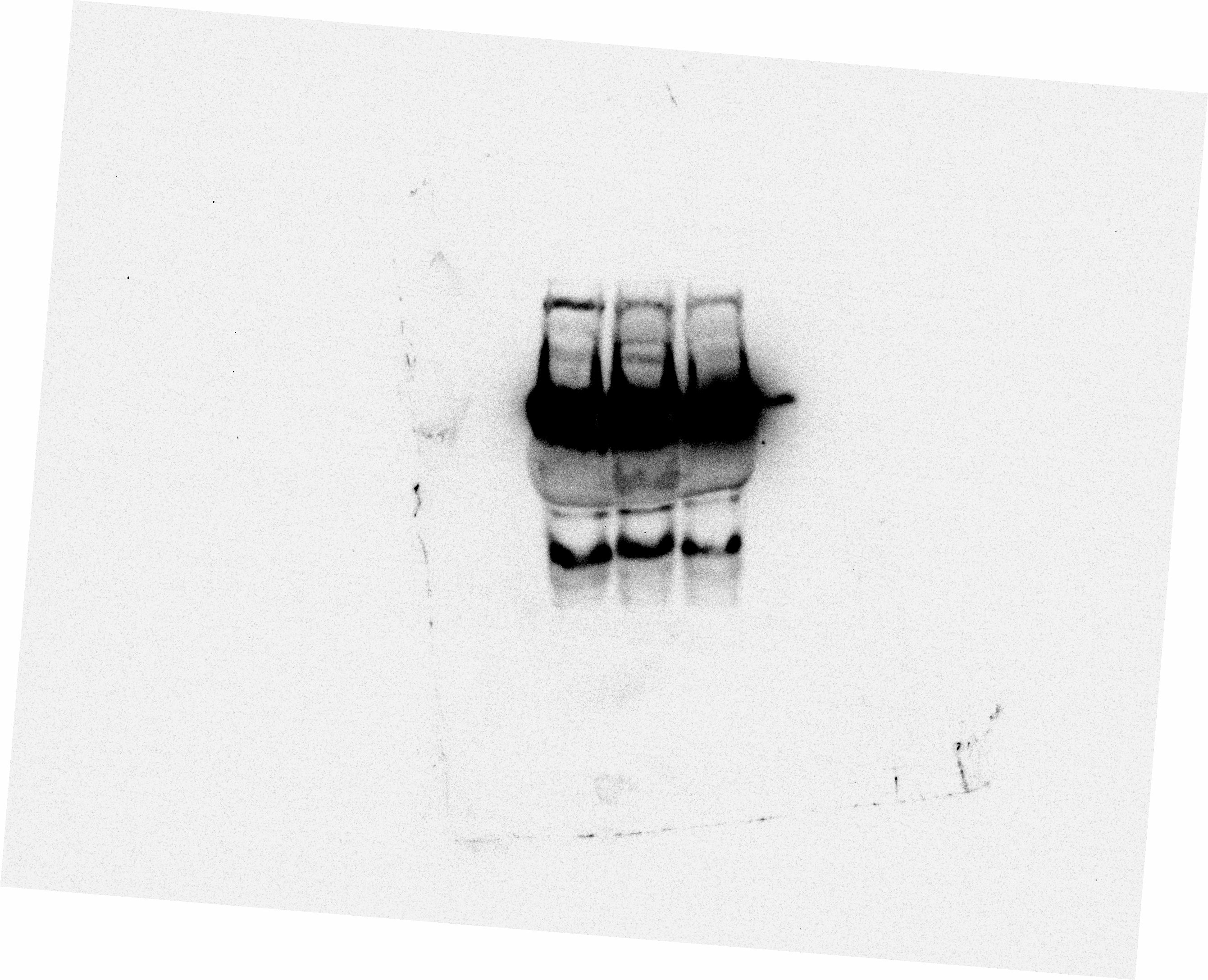


SLC35A2-CDG

Control

SRD5A3-CDG

The original blot of CTSC in plasma


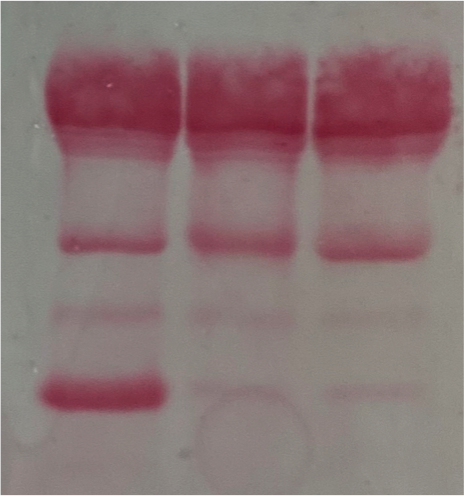


SRD5A3-CDG

SLC35A2-CDG

Control

The original blot of figure 7 showing nitrocellulose membrane stained with ponceau S as a loading marker for CSTC in plasma.
